# Insulin resistance and circadian rhythm of cardiac autonomic modulation

**DOI:** 10.1186/1475-2840-9-85

**Published:** 2010-12-06

**Authors:** Sol M Rodríguez-Colón, Xian Li, Michele L Shaffer, Fan He, Edward O Bixler, Alexandros N Vgontzas, Jianwen Cai, Duanping Liao

**Affiliations:** 1 Department of Public Health Sciences, Penn State University College of Medicine, 600 Centerview Dr. Suite 2200, A210, Hershey, PA, USA; 2 Sleep Research & Treatment Center, Department of Psychiatry. Penn State University College of Medicine. 500 University Drive, Hershey, PA, USA; 3 Department of Biostatistics, University of North Carolina at Chapel Hill, Chapel Hill, NC, USA

## Abstract

**Background:**

Insulin resistance (IR) has been associated with cardiovascular diseases (CVD). Heart rate variability (HRV), an index of cardiac autonomic modulation (CAM), is also associated with CVD mortality and CVD morbidity. Currently, there are limited data about the impairment of IR on the circadian pattern of CAM. Therefore, we conducted this investigation to exam the association between IR and the circadian oscillations of CAM in a community-dwelling middle-aged sample.

**Method:**

Homeostasis models of IR (HOMA-IR), insulin, and glucose were used to assess IR. CAM was measured by HRV analysis from a 24-hour electrocardiogram. Two stage modeling was used in the analysis. In stage one, for each individual we fit a cosine periodic model based on the 48 segments of HRV data. We obtained three individual-level cosine parameters that quantity the circadian pattern: mean (M), measures the overall average of a HRV index; amplitude (Â), measures the amplitude of the oscillation of a HRV index; and acrophase time (θ), measures the timing of the highest oscillation. At the second stage, we used a random-effects-meta-analysis to summarize the effects of IR variables on the three circadian parameters of HRV indices obtained in stage one of the analysis.

**Results:**

In persons without type diabetes, the multivariate adjusted β (SE) of log HOMA-IR and M variable for HRV were -0.251 (0.093), -0.245 (0.078), -0.19 (0.06), -4.89 (1.76), -3.35 (1.31), and 2.14 (0.995), for log HF, log LF, log VLF, SDNN, RMSSD and HR, respectively (all *P *< 0.05). None of the IR variables were significantly associated with Â or θ of the HRV indices. However, in eight type 2 diabetics, the magnitude of effect due to higher HOMA-IR on M, Â, and θ are much larger.

**Conclusion:**

Elevated IR, among non-diabetics significantly impairs the overall mean levels of CAM. However, the Â or θ of CAM were not significantly affected by IR, suggesting that the circadian mechanisms of CAM are not impaired. However, among persons with type 2 diabetes, a group clinically has more severe form of IR, the adverse effects of increased IR on all three HRV circadian parameters are much larger.

## Background

Insulin resistance (IR) is a precursor and a characteristic feature of type 2 diabetes [1]. IR is also associated with higher risk of cardiovascular diseases (CVD) [2,3]. Homeostasis model assessment of IR (HOMA-IR) has been applied to quantify IR in people with or without glucose intolerance, and it has been a reliable tool in the assessment of IR, especially before the clinical diagnosis of type 2 diabetes [4,5].

Heart rate variability (HRV), an index of cardiac autonomic modulation (CAM) [6] is associated with CVD mortality and CVD morbidity in various populations [7-13]. Several epidemiological studies have shown that individuals with IR or increased fasting glucose [14] have increased heart rate [14-16] and reduced HRV [14,16,17]. Galinier et al. demonstrated that patients with hyperinsulinemia or IR had a significant decrease in HRV [18]. In the population-based Atherosclerosis Risk in communities study, a consistent association between IR, metabolic syndrome and impaired CAM has been reported [12,19-23]. However, all of these studies were based on the overall mean levels of HRV.

Several studies have described a circadian pattern of CAM, [24-26] which can be quantified with a cosine periodic regression model consisting of three cosine function parameters: mean (M), amplitude (Â), and acrophase (θ). The cosine function parameter M measures the overall average of a HRV index, the Â measures the amplitude of the oscillation of a HRV index, and the θ measures the clock time when the highest oscillation (amplitude) is reached. Lack of circadian variation of HRV is associated with increased vulnerability to cardiovascular events [27]. However, no study has examined the quantitative effect of insulin resistance on the three major circadian parameters that quantity the circadian pattern of CAM. Therefore, the objective of this study is to examine the effects of IR on the circadian pattern of CAM in a community-dwelling sample of non-diabetics.

## Methods

### Study population

For this report, we used the data collected for the Air Pollution and Cardiac Risk and its Time Course (APACR) study, which we designed to investigate the mechanisms and the time course of the adverse effects of fine particulate matter (PM_2.5_) on cardiac electrophysiology, blood coagulation, and systemic inflammation. Recruitment methods for the APACR study have been published elsewhere [28,29]. All study participants were recruited from communities in central Pennsylvania, primarily from the Harrisburg metropolitan area. The inclusion criteria for the study included nonsmoking adults, ≥ 45 years old, who had not been diagnosed with severe cardiac problems (defined as diagnosed valvular heart disease, congenital heart disease, acute myocardial infarction or stroke within 6 months, or congestive heart failure). Approximately 75% of the individuals who were contacted and who met our inclusion criteria were enrolled in the APACR study. Our targeted sample size was 100 individuals, and we enrolled and examined 102 individuals in the APACR study.

Study participants were examined in the General Clinical Research Center (GCRC) during the morning hours of Day 1, between 8:00 AM and 10:00 AM. After administering the informed consent, the participants completed a health history questionnaire. A trained research nurse measured seated blood pressure three times, height, weight, and drew 50 ml of blood for blood biomarker assays according to the blood sample preparation protocols. A trained investigator connected the PM_2.5 _and Holter electrocardiography (ECG) recorders. Participants were given an hourly activity log to record special events that occurred during 24 hours, including outdoor activities, exposure to traffic on the street, travel in an automobile, and any physical activities. Before participants were released, they received detailed instructions on how to operate both monitors. The entire session lasted approximately 1 hour. The next morning (Day 2), the participants came back to the GCRC to disconnect the PM_2.5 _and Holter monitors, return the completed activity log, have another 50 ml of blood drawn, and provide a urine sample. Then, an exercise echocardiography was performed on each participant according to a standardized protocol (Bruce protocol) to measure the participant's ventricular function and structure. The entire Day 2 session lasted approximately 1 hour and 45 minutes. The study protocol was approved by the Penn State University College of Medicine Institutional Review Board (IRB). All participants gave written informed consent prior to their participation in the study. Each participant was reimbursed with $50.00, a breakfast certificate in the hospital cafeteria, and the mileage of the transportation required for participating in the study.

### Assessment of insulin resistance: insulin, glucose, and HOMA-IR

During the Day 1 visit, after an overnight fast, blood was drawn from each participant. Fasting glucose and insulin were measured by the Penn State College of Medicine GCRC central laboratory. HOMA-IR was calculated as [fasting insulin (μU/mL) Ø fasting glucose (mmol/L)/22.5], with higher HOMA-IR values indicative of more IR [5].

### Continuous Holter ECG recording

#### Continuous ambulatory ECG

A high-fidelity (sampling frequency 1,000 Hz) 12-lead HScribe Holter System (Mortara Instrument, Inc., Milwaukee, WI) was used to collect the 24-hour Holter beat-to-beat ECG data. The high-fidelity ECG significantly increases the resolution and enhances the accuracy of various wave form measurements. All Holter recordings started between 9:00 AM and 10:00 AM. The Holter ECG data were scanned to a designated computer for offline processing by an experienced investigator using specialized SuperECG software (Mortara Instrument, Inc.). The standardized operation procedures (SOPs) for the APACR study developed by the study investigators were followed rigorously in the data collection, offline processes, HRV analysis and interpretation processes. Briefly, the Holter ECG Data Collection and Analysis Procedures were followed to prepare, hook up, calibrate, and start the Holter digital recorder. After 24 hours of recording, a trained investigator followed the SOP to retrieve and archive the beat-to-beat ECG data for offline processing. The main objective of the offline processing was to verify the Holter-identified ECG waves and to identify and label additional electronic artifacts and arrhythmic beats in the ECG recording. Finally, a single research investigator performed beat-to-beat HRV analysis using the normal beat-to-beat RR interval data.

### Cardiac autonomic modulation (CAM) measures

The entire 24-hour normal beat-to-beat RR interval data were divided into 30-minute segments of RR data. Thus, each individual provided 48 segments of 30-minute RR data. The RR data for HRV analysis were processed according to current recommendations [9]. Within each segment, any RR interval <400 ms, >2000 ms, or where the ratio from two adjacent RR intervals was <0.80 or >1.20 were excluded from the HRV analysis. The time- and frequency-domain HRV analysis were performed on the remaining normal RR interval data if the total length of such normal RR intervals was greater than 20 minutes (67% of original data), using the HRV Analysis Software v1.1 [30]. When performing frequency-domain HRV analysis, we used Fast Fourier Transformation (FFT). Briefly, the adjacent RR interval data were interpolated using a piecewise cubic spline approach, with a 2 Hz sampling rate. The FFT was performed on the equidistantly interpolated RR time series. We used a second order polynomial model to remove the slow non-stationary trends of the HRV signal. The following HRV indices were calculated as measures of CAM: standard deviation of all RR intervals (SDNN, ms), square root of the mean of the sum of the squares of differences between adjacent RR intervals (RMSSD, ms), power in the high frequency range (0.15-0.40 Hz, HF), power in the low frequency range (0.04-0.15 Hz, LF), the ratio of LF to HF (LF/HF), and in the very low frequency power (0.00-0.04 Hz, VLF). Following current recommendations [9], we performed logarithmic transformations on HF and LF prior to statistical analysis.

### Covariables

A standardized questionnaire administered on Day 1 to the participants was used to collect the following individual-level information: **(1) **demographic variables, including age, race, sex, and highest education level; **(2) **medication uses, including anti-hypertensive medication and glucose lowering medications; and **(3) **physician diagnosed chronic disease history, including coronary heart disease (e.g., revascularization procedures and myocardial infarction), hypertension, and diabetes. Body weight and standing height were obtained in the morning of Day 1 and were used to calculate body mass index (BMI) as weight (kg)/height (m^2^). Seated blood pressure was obtained three times after 5 minutes of resting. The averages of the second and third measures of seated systolic and diastolic blood pressures were used to represent a participant's blood pressure levels. Hypertension was defined as systolic blood pressure ≥ 140 mmHg, diastolic blood pressure ≥ 90 mmHg, or physician diagnosed hypertension and currently using anti-hypertensive medication. History of CVD was defined as having a physician diagnosed history of myocardial infarction or coronary arterial revascularization procedures. Type 2 diabetes was defined as fasting glucose > 126 mg/dL, physician diagnosed type 2 diabetes, or currently using glucose lowering medication.

### Statistical analysis

From the 102 individuals, we excluded 8 participants with type 2 diabetes. As a result, this report used the data from the remaining 94 individuals. Each individual contributed up to 48 segments of 30-minute RR interval data. After excluding segments where the total length of RR interval data was less than 20 minutes, we analyzed 4404 segments of 30-minute HRV data from 94 individuals.

A two-stage analysis was performed to assess the relationship between IR measures and the circadian pattern of HRV. At the first stage, for each individual we fit the HRV data based on all available 30-minute segments to a cosine periodic regression model [31] using nonlinear least squares: HRV_i_(t) = M_i _+ A_i_•cos [2π •(t-θ_i_)/T] + ε_i_, i = 1, ..., 94, in which M_i _is the daily average of HRV of the i^th ^subject, A_i _is the amplitude of HRV of the i^th ^subject around M_i_, t is the time-specific segment order number, T is the total number of 30-minute segments in 24 hours, θ_i _is the acrophase (the lag from the reference time point (9:00 AM) to the time of the zenith of the cosine curve fit to the data of the i^th ^subject), and ε_i _is the error term of the i^th ^subject. One unit of *t *corresponds to 30 minutes, with 1 indicating 9:00 AM to 9:30 AM, 2 indicating 9:30 AM to 10:00 AM, etc. Thus, from the above described cosine model, we obtained the estimated individual-level cosine periodic regression parameters, namely the M, Â, and θ to quantify the periodicity of the HRV variables. At the second stage, we used random-effects meta-analyses to obtain overall estimates of M, Â and θ, and their 95% confident intervals (CIs) to assess the associations between IR and the three components of the circadian pattern of HRV [32]. Because HOMA-IR and fasting insulin were not normally distributed, we used a logarithmic transformation for these two IR variables before performing the analysis. To visualize the impact of elevated IR on the three circadian parameters, we plotted the population level circadian pattern of HF, and superimposed the circadian pattern predicted HF from one standard deviation increase in HOMA-IR. To confirm the associations between the IR and the means from the random-effects meta-analysis models, we also applied linear mixed-effects models, specifying a first-order autoregressive covariance structure, to assess the associations between HRV variables and HOMA-IR using 24-hour data, analyzing the daytime (9 AM to 9 PM) and nighttime (9 PM to 9 AM next day) data separately. This model will also enable us to examine whether IR affect daytime and nighttime autonomic modulation differently. In this approach we ignored the assumption of the specific cosine form for the data and treated the HRV variables from each 30-minute segment as repeated measures. All analyses were performed using SAS version 9.1 software (SAS Institute Inc., Cary, NC, USA).

## Results

### Study population characteristics

The main characteristics of the study population are shown in Table 1. From the first 102 APACR study enrollees, we excluded 8 individuals who had a history of type 2 diabetes. The sample size for this analysis was 94 individuals. The mean (SD) age of the entire study population was 56.5 (7.8) years old, with 37% male and 77% white. The prevalence of CVD was 7.4%. In addition, the mean (SD) for BMI (kg/m^2^), insulin (μU/mL), glucose (mg/dL), and HOMA-IR of the entire cohort were 27.1 (4.9), 6.6 (6.7), 84.4 (11.3), and 1.45 (1.73) respectively. The means (SD) for the HRV indices: log HF, log LF, LF/HF ratio, log VLF, SDNN, RMSSD, and HR were 4.4 (0.9), 5.4 (0.8), 3.8 (2.2), 6.9 (0.6), 61.8 (18.4), 29.6 (14.8), and 76.5 (9.7), respectively.

**Table 1:** The study population demographic characteristics and summaries of HRV indices.

*Demographics*	*All subjects (n = 94)*
Age (years)	56.5 (7.8)
Gender (% male)	37.2
Race (% white)	76.6
BMI (kg/m^2^)	27.1 (4.9)
College or higher (%)	78.2
Cardiovascular disease (%)	7.4
Hypertension (%)	33.0
Systolic BP (mmHg)	122.0 (15.4)
Diastolic BP (mmHg)	75.0 (9.3)
Insulin (μU/mL)	6.6 (6.7)
Log insulin (μU/mL)	1.5 (0.8)
Glucose (mg/dL)	84.4 (11.3)
HOMA-IR	1.45 (1.73)
Log HOMA-IR	-0.030 (0.86)
HRV indices*:	
Log of HF (ms^2^)	4.4 (0.9)
Log of LF (ms^2^)	5.4 (0.8)
LF/HF ratio	3.8 (2.2)
Log of VLF (ms^2^)	6.9 (0.6)
SDNN (ms)	61.8 (18.4)
RMSSD (ms)	29.6 (14.8)
Heart rate (BPM)	76.5 (9.7)

Abbreviations: BMI, body mass index; BP, blood pressure; BPM, beats per minute; HF, high frequency power; HOMA-IR, homeostasis model assessment of insulin resistance; HRV, heart rate variability; LF, low frequency power; Log, logarithm; RMSSD, square root of the mean of the sum of the squares of differences between adjacent RR intervals; SDNN, standard deviation of all RR intervals; VLF, very low frequency power.

Data are expressed as mean (SD) or percentage.

*: Overall average of the intra-subject means.

### Circadian pattern of HRV variables

The estimated means of the three cosine periodic regression parameters and their 95% CIs for each of the HRV indices are presented in Table 2. The acrophases of log HF and RMSSD (both are reflective of the vagal modulation) were very similar, 4:00 AM and 4:15 AM, respectively. The acrophases of log LF and SDNN (both are reflective of sympathetic and vagal modulation) were also very similar - the point estimates are around 6:00 AM ± 45 minutes. The acrophase of log VLF was 8:00 AM, LF/HF ratio was 6:00 PM, and that of heart rate was 2:00 PM. The tests of zero amplitude were highly significant (*P *value < 0.0001) for every HRV variable, suggesting a significant circadian variation.

**Table 2:** Estimated means of the three cosine periodic regression parameters and their 95% CIs for each HRV index.

	*Cosine periodic parameter*		
	
*HRV index*	*Mean (95% CI)*	*Amplitude (95% CI)*	*Acrophase (95% CI)^†^*
Log HF (ms^2^)	4.36 (4.17-4.54)	0.60 (0.53-0.67)*	4:00(3:00-5:00) AM
Log LF (ms^2^)	5.36 (5.21-5.52)	0.44 (0.39-0.49)*	5:30(4:30-6:45) AM
LF/HF ratio	3.74 (3.33-4.15)	1.16 (0.98-1.34)*	6:00(4:30-7:15) PM
Log VLF (ms^2^)	6.95 (6.83-7.07)	0.39 (0.35-0.44)*	8:00(7:00-9:00) AM
SDNN (ms)	60.8 (57.2-64.4)	10.5 (9.3-11.7)*	6:45(5:45-7:30) AM
RMSSD (ms)	28.4 (25.7-31.0)	7.24 (6.10-8.37)*	4:15(3:15-5:15) AM
Heart rate (BPM)	76.6 (74.6-78.6)	10.8 (9.8-11.7)*	2:00(1:45-2:30) PM

Abbreviations: BPM, beats per minute; CIs, confidence intervals; HF, high frequency power; HRV, heart rate variability; LF, low frequency power; Log, logarithm; RMSSD, square root of the mean of the sum of the squares of differences between adjacent RR intervals; SDNN, standard deviation of all RR intervals; VLF, very low frequency power.

*: *P *value < 0.0001 for zero amplitude test of the HRV indices.

*†: *Calculated as the unit to peak ± 2 standard errors, and translated into clock time.

### Insulin resistance and HRV circadian pattern

The associations between the IR measurements and cosine parameters of the circadian pattern (M, Â, and θ) estimated from the entire sample using random-effects meta-analysis models are presented in Table 3. Two different random-effects meta-analysis regression models were reported: Model 1, unadjusted; Model 2, adjusted for age sex, race, hypertension, and history of CVD. In general, higher levels of IR, especially IR measured by HOMA-IR, was significantly associated with lower M of all HRV indices and higher HR in both unadjusted and multivariable adjusted models, except that LF/HF ratio was not significantly associated with any measure of IR. In this healthy non-diabetes sample, IR was not significantly associated with the Â or θ of any HRV indices. Log VLF was also included in Table 3 as a HRV variable that may be reflective of nocturnal sympathetic hyperactivity. The association between IR measures and VLF is very similar to that of HF and LF - elevated insulin resistance is only associated with lower mean levels of VLF, but not with the amplitude or the acrophase.

**Table 3:** The association between cosine parameters of circadian pattern with insulin resistance measures based on random-effects meta-analysis

			*HRV index*													
			
			*Log HF (ms^2^)*		*Log LF (ms^2^)*		*LF/HF ratio*		*Log VLF(ms^2^)*		*SDNN (ms)*		*RMSSD (ms)*		*Heart Rate (bpm)*	
			*Beta*	*SE*	*Beta*	*SE*	*Beta*	*SE*	*Beta*	*SE*	*Beta*	*SE*	*Beta*	*SE*	*Beta*	*SE*
Log HOMA-IR	M1	M	-0.25	0.09**	-0.24	0.08**	0.11	0.21	-0.19	0.06**	-4.89	1.76**	-3.35	1.31**	2.14	0.99*
		Â	0.04	0.03	-0.01	0.03	0.15	0.09	0.01	0.02	-0.19	0.61	-0.34	0.59	-0.28	0.48
		θ	0.11	0.91	1.33	1.12	-1.56	1.35	0.22	0.91	-0.60	0.91	-1.24	1.00	0.47	0.33
	M2	M	-0.28	0.10**	-0.28	0.08**	0.08	0.22	-0.23	0.07**	-5.90	1.90**	-3.50	1.45*	2.57	1.11*
		Â	0.01	0.04	-0.01	0.03	0.08	0.10	0.03	0.03	-0.002	0.67	-0.61	0.67	-0.16	0.54
		θ	0.22	1.04	1.04	1.26	-0.07	1.42	-0.02	1.01	-0.22	1.02	-1.14	1.12	0.26	0.37
																
Log insulin	M1	M	-0.23	0.09*	-0.23	0.08**	0.05	0.21	-0.18	0.06**	-4.41	1.75**	-3.03	1.30*	1.94	1.00
		Â	0.04	0.03	-0.02	0.03	0.14	0.09	0.01	0.02	-0.21	0.60	-0.18	0.58	-0.09	0.48
		θ	-0.40	0.94	0.91	1.14	-1.84	1.33	0.13	0.92	-0.87	0.92	-1.84	1.01	0.46	0.33
	M2	M	-0.25	0.10**	-0.26	0.08**	0.04	0.21	-0.20	0.06**	-5.21	1.89**	-3.15	1.44*	2.18	1.12*
		Â	0.02	0.04	-0.01	0.03	0.08	0.09	0.02	0.02	0.04	0.66	-0.35	0.65	0.07	0.54
		θ	-0.41	1.07	0.47	1.27	-0.44	1.39	-0.26	1.02	-0.70	1.03	-1.82	1.13	0.24	0.36
																
Glucose	M1	M	-0.20	0.09*	-0.17	0.08*	0.21	0.21	-0.16	0.06**	-4.05	1.79*	-2.31	1.34	1.04	1.01
		Â	-0.01	0.03	0.02	0.03	0.10	0.09	0.03	0.02	0.42	0.61	-0.81	0.59	-0.83	0.47
		θ	1.36	0.90	1.43	1.12	0.92	1.35	-0.21	0.91	0.07	0.92	1.42	1.02	0.08	0.34
	M2	M	-0.16	0.10	-0.18	0.08*	0.08	0.22	-0.18	0.07**	-4.41	1.93*	-1.84	1.48	1.79	1.12
		Â	-0.04	0.04	0.01	0.03	0.05	0.10	0.05	0.02	0.32	0.67	-1.25	0.66	-0.88	0.53
		θ	1.85	1.02	1.82	1.24	1.91	1.39	0.32	0.98	1.19	0.10	1.87	1.13	-0.05	0.37

Abbreviations: Â, amplitude; BPM, beats per minute; HF, high frequency power; HOMA-IR, homeostasis model assessment of insulin resistance; HRV, heart rate variability; LF, low frequency power; Log, logarithm; M, mean; RMSSD, square root of the mean of the sum of the squares of differences between adjacent RR intervals; SDNN, standard deviation of all RR intervals; VLF, very low frequency power; θ, acrophrase time.

Models: M1, unadjusted; M2, adjusted for age, sex, race, hypertension, and cardiovascular diseases.

*Beta *= one SD increase of insulin resistance measurements. *, and **, *P *< 0.05, and *P *< 0.01, respectively.

**Figure 1:**
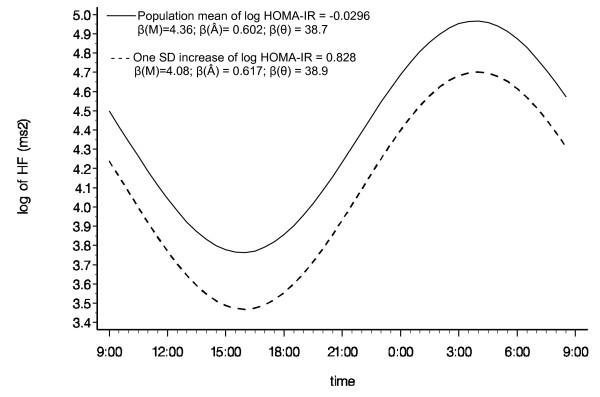
**Multivariable adjusted (Model 2) cosine periodic model estimates for log HF over the clock time at the population mean and one SD above the mean in log HOMA-IR**. Abbreviations: Â, amplitude; HF, high frequency power; HOMA-IR, homeostasis model assessment of insulin resistance; log, logarithm; M, mean; SD, standard deviation; θ, acrophrase time.

The circadian variation of Log HF calculated from the entire population is graphically presented in Figure 1. The predicted circadian pattern of Log HF due to a one SD increase in HOMA-IR is superimposed in Figure 1 to graphically illustrate the impact of elevated IR on the circadian pattern of log HF. It clearly show, similar to the numeric numbers in Table 3, that one SD increase in IR is associated with lower overall mean [β (M)] of Log HF, but not with the other two circadian parameters (Â and θ).

We also performed additional analyses examining the associations between HOMA-IR and HRV cosine parameters of circadian pattern in 8 individuals with physician-diagnosed type 2 diabetes. These analyses were preformed in an unadjusted model because small sample size. The results are presented in Table 4. Compared to that regression coefficients in Table 3 Model 1, the magnitude of effect due to higher Log HOMA-IR on the mean (M), amplitude (Â), and acrophase time (θ), are much larger among persons with type 2 diabetes, a group that clinically have more severe form of insulin resistance. Because of the small sample size, most of the statistical tests of the regression coefficients in Table 4 did not reach traditional significant level (*P *value < 0.05).

**Table 4:** The association between cosine parameters of circadian pattern with Log HOMA-IR based on random-effects meta-analysis of 8 physician diagnosed type 2 diabetes individuals.

			*HRV index*											
			
			*Log of HF (ms^2^)*		*Log of HF (ms^2^)*		*SDNN (ms)*		*RMSSD (ms)*		*LF/HF Ratio*		*Heart Rate (BPM)*	
			*Beta*	*SE*	*Beta*	*SE*	*Beta*	*SE*	*Beta*	*SE*	*Beta*	*SE*	*Beta*	*SE*
Log HOMA-IR	M1	M	-0.57	0.29	-0.48	0.28	-6.14	8.12	-3.20	4.56	0.28	0.27	0.30	3.39
		Â	-0.03	0.15	-0.003	0.12	-0.35	2.39	-2.30	1.24	0.04	0.17	-2.19	1.21
		θ	7.56	3.64	4.36	4.15	6.99	3.32	9.04	3.49*	2.51	4.28	0.97	1.63

Abbreviations: Â, amplitude; BPM, beats per minute; HF, high frequency power; HOMA-IR, homeostasis model assessment of insulin resistance; HRV, heart rate variability; LF, low frequency power; Log, logarithm; M, mean; RMSSD, square root of the mean of the sum of the squares of differences between adjacent RR intervals; SDNN, standard deviation of all RR intervals; VLF, very low frequency power; θ, acrophrase time.

M1: unadjusted model; *Beta*: one SD increase of Log HOMA-IR.

* = *P *< 0.05

Ignoring the specific cosine form for the data and treating HRV variables from each 30-minute segment as repeated measures, the regression coefficients, SE, and *P *value relating HOMA-IR and HRV indices according to daytime (9 AM to 9 PM) and nighttime (9 PM to 9 AM next day) are presented in Table 5. In general, these results confirmed that higher IR is associated with lower HRV indices, and the patterns of association are similar daytime and night time.

**Table 5:** The regression coefficients (β) estimates and standard error (SE) of Log-HOMA-IR on HRV indices using 24-hour data, daytime (9 AM to 9 PM) and nighttime (9 PM to 9 AM next day) data separately from linear mixed-effects models analysis.

			*HRV index*											
			
			*Log of HF (ms^2^)*		*Log of LF (ms^2^)*		*SDNN (ms)*		*RMSSD (ms)*		*LF/HF Ratio*		*Heart Rate (bpm)*	
			
			*Beta*	*SE*	*Beta*	*SE*	*Beta*	*SE*	*Beta*	*SE*	*Beta*	*SE*	*Beta*	*SE*
Log HOMA-IR	M 1	24-h	-0.23	0.028**	-0.24	0.021**	-3.58	0.31**	-1.93	0.34**	0.086	0.062	2.85	0.41**
		day	-0.26	0.034**	-0.22	0.027**	-3.44	0.38**	-1.86	0.35**	0.35	0.083**	2.80	0.60**
		night	-0.19	0.043**	-0.26	0.030**	-3.73	0.49**	-1.87	0.58**	-0.18	0.092*	2.88	0.52**
	M 2	24-h	-0.32	0.028**	-0.29	0.021**	-4.85	0.33**	-2.64	0.37**	0.27	0.066**	3.34	0.46**
		day	-0.33	0.035**	-0.28	0.029**	-4.31	0.39**	-2.32	0.37**	0.52	0.086**	3.21	0.68*
		night	-0.30	0.040**	-0.31	0.032**	-5.40	0.52**	-2.78	0.62**	0.011	0.10	3.36	0.58**

Abbreviations: BPM, beats per minute; HF, high frequency power; HOMA-IR, homeostasis model assessment of insulin resistance; HRV, heart rate variability; LF, low frequency power; Log, logarithm; M, mean; RMSSD, square root of the mean of the sum of the squares of differences between adjacent RR intervals; SDNN, standard deviation of all RR intervals.

Models: M1, unadjusted; M2, adjusted for age, sex, race, hypertension, and cardiovascular diseases.

*Beta *= one SD increase of Log HOMA-IR.

*, and **, *P *< 0.05, and *P *< 0.001, respectively.

## Discussion

The estimated means and their 95% CIs of the three cosine periodic regression parameters presented in Table 2 clearly show a distinctive 24-hour based circadian rhythm of HRV indices. For instance, using Log HF as an example, a recognized HRV marker of parasympathetic modulation, had an overall mean of 4.36 ms^2 ^and reached the acrophase (highest amplitude of Â = 0.60 ms^2^) around 4:00 AM. Furthermore, the tests of zero amplitude were highly significant (*P *value < 0.0001) for every HRV variable, suggesting a significant circadian variation. The mean (M), amplitude (Â), and acrophase time (θ) together define the circadian pattern of HRV, which is reflective of the balance of two opposing autonomic modulation branches, namely parasympathetic and sympathetic modulations.

Past epidemiological studies performed in different populations investigating the relationship between IR and the overall mean levels of HRV indices have shown that elevated IR has a significant adverse effect on the overall mean levels of CAM [17,33]. Pikkujämsä et al. demonstrated that subjects with IR syndrome have reduced HRV indices [17]. In addition, Perciaccante et al. confirmed that impaired autonomic activity was present in IR patients [33]. On the other hand, other studies have shown that individuals without type 2 diabetes or offspring of type 2 diabetic persons, even when their glucose was in the normal range, had lower HRV indices [14,16,34]. Stein et al. found that in non-diabetic individuals with normal fasting glucose levels, higher glucose levels [14] and higher HOMA-IR [16] were associated with faster heart rate and lower HRV. In addition, Fiorentini et al. showed that higher HOMA-IR was associated with autonomic impairment among the offspring of individuals with type 2 diabetes, even when they had no history of type 2 diabetes, normal glucose tolerance test, and no history of hypertension [34]. Similar to these previous findings, in this community-dwelling sample of healthy non-smokers without type 2 diabetes, we found that a higher level of IR is significantly associated with lower mean levels of HRV. The pattern of these associations remained significant after adjustment for age, sex, race, hypertension, and history of CVD. This conclusion is also supported in the analysis treating HRV variables from each 30-minute segment as repeated measures, and stratified according to daytime (9 AM to 9 PM) and nighttime (9 PM to 9 AM next day). Moreover, the daytime and night time stratified model suggest a similar daytime and nighttime association between IR and CAM.

To our knowledge, none of the previously published studies investigated the effects of IR on the other two parameters of HRV circadian pattern, the amplitude and the acrophase. In this study, we used a two-stage analytic approach to first obtain individual-level cosine function parameters (M, Â, and θ) from 24 hours of HRV data, and then used a random-effects meta-analysis regression model to investigate the impact of IR on each of the circadian cosine parameters. This approach allows us to estimate the impact of IR, not only on overall mean levels of HRV variables as in previously published studies, but it also enabled us to examine the impact of IR on the amplitude and acrophase time of HRV. In our data obtained from obviously healthy non-diabetics and non-smokers, IR was not associated with impaired circadian variation of HRV as suggested by lack of association between amplitude and acrophase time of HRV index and IR.

In humans, circadian rhythms in blood pressure [35,36], and in heart rate have been intensively studied, mainly due to the increased cardiovascular death reported during the morning hours [37,38]. There are a large number of different biological circadian rhythms, including numerous aspects of cardiovascular functions [39]. These are generated by an endogenous oscillator, composed of a central clock which resides in the hypothalamic suprachiasmic nucleus (SCN), and peripheral clock in peripheral tissues [40]. These two have an important role in the timing and organization/coordination of sleep with other physiological rhythms, such as the balance of autonomic modulation [41]. The central clock is composed of self-sustaining single cell circadian oscillators which produce circadian signals [40,42]. These oscillators are not precisely 24 hours, so they adjust to the external light/dark signal from the retinohypothalamic tract [40,42]. These signals give information about the 24-hour day pattern, the relative proportion of light/dark, and the amplitude of light intensity that tracks the seasons [43]. Most of the peripheral tissue cells have similar oscillations like the SCN [44].

Various animal studies have examined the impact of disruption clock genes [45-48]. Turek et al. showed that homozygous Clock mutant mice had a loss of sense mutation, lost circadian rhythmicity, became obese, and developed hyperglycemia among other health problems [32]. Rudic et al. showed that mutations in Bmal and Clock modified circadian variation in glucose and triglycerides and influenced the development of IR [46]. Marcheva et al. found that phase of oscillation of islet genes involved in growth, glucose metabolism and insulin signaling is delayed in circadian mutant mice, and both Clock and Bmal1 mutants show reduce insulin secretion, and impaired glucose tolerance [47].

Researchers have been concerned over the disruption of these circadian rhythms in the human clock, which regulate the metabolism in time with day length [49,50]. For example, glucose and insulin have been shown to exhibit diurnal variation in humans [50]. The normal physiological variation of these variables between day and night could be lost (with an increase over the 24 hours) contributing to the development of CVD [50] or IR.

Earlier studies have investigated the circadian rhythm of HRV in humans [15,51,52]. Huikuri et al. observed that the circadian rhythm of HRV (measured from a 24-hour ECG) in healthy subjects had a maximum occurring early in the morning before waking up, reflecting higher parasympathetic tone, but decreased abruptly during the hours after waking [51]. Malpa et al. found that HRV variation over 24 hours in alcoholics and persons with diabetes was significantly reduced compared to normal controls [25]. Huikuri et al. demonstrated that there was a circadian pattern of HRV in both survivors of cardiac arrest and controls, with acrophase occurring in the morning hours before arousal, followed by a decrease in HRV after arousal; however there was low vagal modulation in survivors of cardiac arrest in the morning [52].

To our knowledge, the effects of IR on the circadian rhythm (amplitude and acrophase time) of HRV have not been reported in the literature. The aim of this study was to investigate the association between IR and the circadian pattern of CAM assessed by three circadian cosine parameters of HRV. In our data, higher levels of IR, as assessed by HOMA-IR, fasting insulin, or fasting glucose, were significantly associated with lower mean levels of HRV. However, IR in this obviously healthy non-diabetic and nonsmoking population sample was not associated with impaired circadian variation of HRV indices, i.e., lower amplitude and acrophase time of any HRV index. This lack of significant effects on the circadian pattern of HRV variation, indicates that the burden of IR in our healthy study population is not large enough to exert its effects yet. We speculate that with prolonged burden of elevated subclinical IR, possibly long before full manifestation of clinical diabetes, all three circadian parameters of CAM may be adversely affected. This is supported by the analysis of the associations between Log HOMA-IR and HRV cosine parameters of circadian pattern in 8 individuals with physician-diagnosed type 2 diabetes (Table 4). Specifically, the adverse effects of increased IR on all three HRV circadian parameters, namely the mean (M), amplitude (Â), and acrophase time (θ), are much larger among persons with type 2 diabetes, a group that clinically have more severe form of IR.

Future longitudinal studies are needed to investigate this hypothesis. From a practical stand point, the results of lack of effects on the circadian pattern of HRV also suggest that early interventions to enhance insulin sensitivity, such as physical activity, may be helpful to preserve the circadian pattern of CAM.

A few limitations of this study should be recognized. First, our cross-sectional data limited our ability to establish a temporal relationship. Thus, our results support the need for a longitudinal study to investigate the complex relationships between IR and CAM assessed by circadian variation of HRV indices. Second, we only collected data from nonsmokers who did not have physician diagnosed heart disease in the 6 months prior to participating in our study. Therefore, our findings cannot be generalized to smokers or individuals with recent acute cardiac events. However, there are several strengths worth mentioning. First, the characteristics of this population were very similar to those in the general population. We analyzed several variables that are characteristics of IR, including insulin, glucose, and HOMA-IR. These variables, especially HOMA-IR, are considered reliable quantitative measures of IR before clinical diabetes [4,5]. Second, distinct from previous publications, which primarily analyzed short time HRV variables, [17,53] we utilized cosine periodic functions to fit individual-level models to the 24 hours of HRV data to estimate the individual-level mean, as well as amplitude and crescent time, of each HRV variable. This enabled us to examine not only the impact of various independent variables, such as HOMA-IR, on the mean levels of HRV variables, but also allowed us to examine the impact of such variables on the other circadian cosine parameters of the HRV indices.

## Conclusion

In this cross-sectional study of community dwelling non-smokers, after excluding the few individuals with a history of diabetes, subclinical IR only exhibited significant adverse effects on the mean levels of HRV. The subclinical IR in this sample did not show an adverse impact on the amplitude and the acrophase time of the circadian cosine parameters. It might be anticipated that with fully manifested diabetes, the consequence of prolonged IR, the entire circadian variation of CAM will be adversely affected. From a practical stand point, these data suggest the potential for early intervention to improve insulin sensitivity and to preserve the circadian pattern of CAM, which is protective from cardiovascular adverse events.

## Competing interests

The authors declare that they have no competing interests.

## Authors' contributions

All authors listed on the manuscript participated in the design and coordination of the study and made substantial contribution to the intellectual content of the project to be included as authors. They also read and approved the final manuscript.
